# Recognizing the Top Peer Reviewers in 2024 for the *Journal of Virology*

**DOI:** 10.1128/jvi.00539-25

**Published:** 2025-05-20

**Authors:** Felicia Goodrum, Stacey Schultz-Cherry

**Affiliations:** 1 University of Arizona College of Medicine 12216 , Tucson, Arizona, USA; 2 Infectious Diseases, St. Jude Children’s Research Hospital 5417, Memphis, Tennessee, USA

**Keywords:** microbiology, peer review, virology

## EDITORIAL

The *Journal of Virology* (JVI) remains the leading journal in virology, with 78,636 citations in 2023, reaffirming its status as the most cited journal in the Virology category, according to the latest *Journal Citation Reports* (Clarivate, 2024). This excellence is derived directly from JVI authors, reviewers, editors, and staff. The editors, editorial board members, and reviewers are to be commended for their hard work in ensuring a smooth review process that is constructive, rigorous, fair, and objective, as well as expedient. The median time from manuscript submission to an editor’s ﬁrst peer review decision letter now stands at 29 days. We thank all the JVI reviewers over the past year. We would especially like to thank the following 30 reviewers, whose service to JVI during the past year was exceptional in the total number of manuscripts reviewed, submitting reviews on time >80% of the time with an average of 11 days or less. All of these reviewers completed 10 or more reviews in 2024. A special shout-out to the reviewers who were also on the 2023 Top Reviewer list (individuals listed in bold).

Matthew Aliota, University of Minnesota Twin Cities

Alan Barrett, University of Texas Medical Branch

**David Barton, University of Colorado School of Medicine**

George A. Belov, University of Maryland, College Park

**Nora M. Chapman, University of Nebraska Medical Center**

**Saurabh Chattopadhyay, University of Kentucky**

Young Ki Choi, Institute for Basic Science

**Xufang Deng, Oklahoma State University**

Anthony R. Fehr, The University of Kansas

**Zongdi Feng, The Research Institute at Nationwide Children’s Hospital**

**Douglas Gladue, Seek Labs**

Ju-Tao Guo, Baruch S. Blumberg Institute

Syed Saif Hasan, University of Maryland School of Medicine

**Ronald M. Iorio, University of Massachusetts Medical School**

**Dong-Yan Jin, University of Hong Kong**

**Clinton Jones, Oklahoma State University**

Kylene Kehn-Hall, Virginia Polytechnic Institute and State University

**Florian Krammer, Icahn School of Medicine at Mount Sinai**

Shinji Makino, The University of Texas Medical Branch

Richard K. Plemper, Georgia State University

Stefan H. Pöhlmann, Deutsches Primatenzentrum GmbH - Leibniz-Institut fur

Primatenforschung

Zhaohui Qian, Chinese Academy of Medical Sciences & Peking Union Medical College

**Bert K. Rima, Queen’s University Belfast**

Raymond R. Rowland, Kansas State University

Isabel Sola, Centro Nacional de Biotecnologia, CSIC

Kenneth A. Stapleford, New York University Grossman School of Medicine

Ashley St. John, Duke-National University of Singapore

Penghua Wang, University of Connecticut School of Medicine

Xianfang Wu, Cleveland Clinic

**Shuqi Xiao, Lanzhou Veterinary Research Institute**

